# Optimizing efficiency and safety in external beam radiotherapy using automated plan check (APC) tool and six sigma methodology

**DOI:** 10.1002/acm2.12678

**Published:** 2019-08-19

**Authors:** Shi Liu, Karl K. Bush, Julian Bertini, Yabo Fu, Jonathan M. Lewis, Daniel J. Pham, Yong Yang, Thomas R. Niedermayr, Lawrie Skinner, Lei Xing, Beth M. Beadle, Annie Hsu, Nataliya Kovalchuk

**Affiliations:** ^1^ Department of Radiation Oncology Stanford University Stanford CA USA; ^2^ Davidson College Davidson NC USA; ^3^ Department of Radiation Oncology Washington University School of Medicine St. Louis MO USA

**Keywords:** DMAIC methodology, ESAPI script, failure mode and effect analysis (FMEA), physics plan check, safety in radiotherapy, six sigma, treatment planning error

## Abstract

**Purpose:**

To develop and implement an automated plan check (APC) tool using a *Six Sigma* methodology with the aim of improving safety and efficiency in external beam radiotherapy.

**Methods:**

The *Six Sigma* define‐measure‐analyze‐improve‐control (DMAIC) framework was used by measuring defects stemming from treatment planning that were reported to the departmental incidence learning system (ILS). The common error pathways observed in the reported data were combined with our departmental physics plan check list, and AAPM TG‐275 identified items. Prioritized by risk priority number (RPN) and severity values, the check items were added to the APC tool developed using Varian Eclipse Scripting Application Programming Interface (ESAPI). At 9 months post‐APC implementation, the tool encompassed 89 check items, and its effectiveness was evaluated by comparing RPN values and rates of reported errors. To test the efficiency gains, physics plan check time and reported error rate were prospectively compared for 20 treatment plans.

**Results:**

The APC tool was successfully implemented for external beam plan checking. FMEA RPN ranking re‐evaluation at 9 months post‐APC demonstrated a statistically significant average decrease in RPN values from 129.2 to 83.7 (*P *< .05). After the introduction of APC, the average frequency of reported treatment‐planning errors was reduced from 16.1% to 4.1%. For high‐severity errors, the reduction was 82.7% for prescription/plan mismatches and 84.4% for incorrect shift note. The process shifted from 4σ to 5σ quality for isocenter‐shift errors. The efficiency study showed a statistically significant decrease in plan check time (10.1 ± 7.3 min, *P* = .005) and decrease in errors propagating to physics plan check (80%).

**Conclusions:**

Incorporation of APC tool has significantly reduced the error rate. The DMAIC framework can provide an iterative and robust workflow to improve the efficiency and quality of treatment planning procedure enabling a safer radiotherapy process.

## 1 INTRODUCTION

Patient safety and error prevention are essential considerations for external beam radiation therapy (EBRT). Approximately 40% of all EBRT tasks are focused primarily on detecting and fixing errors.^1^ While the error rate per patient is seemingly low,^2^ catastrophic consequences may be caused by the most severe errors, such as incorrect treatment location, incorrect dose, data entry errors, or equipment malfunctions. Thus, the tolerance for such errors must be as low as reasonably achievable. The predominant approach is to use well‐established quality assurance (QA) and quality control (QC) processes to minimize errors prior to treatment delivery.^3^, ^4^ The most typical types of QC/QA processes include a combination of physics plan check, physician plan review, peer‐review chart rounds, pretreatment QA for intensity‐modulated radiation therapy (IMRT), therapist timeouts, and physics weekly chart check.^5^ As the majority of errors in radiotherapy originate in treatment planning,^6^ the physics plan check was found to be the most effective individual QC step in the radiotherapy workflow.^7^ However, its sensitivity to identify a defect is still low: according to Gopan et al., only 38% of errors that could have been detected at the time of physics plan check were actually detected, the remainder 62% went undetected.^8^ As technological advances can make manual verification of treatment plans increasingly challenging, automation and computerization can offer greater effectiveness thereby potentially enhancing safety.^9^

Software with automatic plan verification functionalities based on predefined rules has been developed in several institutions and previously reported.^10^, ^11^, ^12^, ^13^, ^14^, ^15^, ^16^, ^17^ In this work, we applied the *Six Sigma* define‐measure‐analyze‐improve‐control (DMAIC) methodology to develop and implement an automated plan check (APC) tool, aiming at reducing errors stemming from treatment planning. We chose to apply a *Six Sigma* methodology which provides a structured framework to measure and reduce defects in the process and has been successfully employed in other radiation oncology settings.^18^, ^19^, ^20^ To enhance the value of such an APC tool, we used failure mode and effects analysis (FMEA) as the foundation to identify high‐severity and high‐risk priority numbers (RPN) check items and prioritize them in developing our APC tool. Tailored specifically to the authors’ clinic using the eclipse scripting application programming interface (ESAPI, Varian Medical Systems, Palo Alto, CA), the APC tool was built and integrated in the clinical workflow by dosimetrists and physicists. The APC tool was optimized in several cycles to fit the needs of the clinic and make the physics plan check more robust and efficient.

## 2 MATERIALS AND METHODS

### 2.1 Scope of study

Treatment planning and delivery in our department is performed via an integrated ARIA Record and Verify (R&V) and Eclipse Treatment Planning System v13.6 (Varian Medical Systems). On average over 2900 EBRT plans are created a year by 10 dosimetrists across three cancer center sites. There are two web‐based incident learning systems (ILS) in the department: (a) department‐wide “Safety Through Alertness and Reaction” (STAR) system and (b) “Good Catch!” — a simple web‐based form permitting anonymous reporting by dosimetrists, physicists, and therapists. Both ILS are complementary and designed to encourage reporting:

- “STAR” system is a non‐anonymous 22‐item web form open to the whole radiation oncology department created to collect higher‐severity incidents, near misses and workflow issues and notify all the managers in the department. The incidents are then reviewed by a committee with follow‐up root cause analysis.
- “Good Catch!” system is an anonymous four‐item web form open to physicists, dosimetrists and therapists to quickly and anonymously report the lower‐severity near misses and errors. The near misses are then reviewed by a committee and discussed at the interdisciplinary monthly meetings.

In this study, we only evaluated errors or near misses that stemmed from treatment planning and were detected by any of these ILS and reported at physics plan check, therapy plan check, or treatment. A near miss or error was defined as a defect that could have or did result in quality or time loss. An example of a near miss: shift instructions for the therapists contained incorrect shift value but this was caught and corrected by the physicist performing the plan check. On the other hand, an example of an error: incorrect shift instructions for the therapists resulted in delivery of the first fraction at incorrect SSD resulting in 4.8% discrepancy between the planned and delivered dose to the target for the first fraction.

A *Six Sigma* approach using five phases, define‐measure‐analyze‐improve‐control, was undertaken with the goal of reducing the reported treatment planning incidents and improving the physics plan check time efficiency.

#### 2.1.1 Define stage

The *Define* stage was aimed at outlining the overall goals and mapping out a strategy to achieve them. To achieve the goal of reducing treatment planning incidents and improve physics plan check efficiency, the QI team followed a DMAIC methodology and marked out important phases of the project: (a) review the history of reported events, (b) compile the plan check list and identify potential automation opportunities while prioritizing the high‐risk and high‐severity checks using FMEA, (c) develop the APC software, (d) enforce implementation procedures and protocols, (e) create a feedback loop, and (f) analyze the improvements.

#### 2.1.2 Measure stage

The *Measure* stage was aimed at understanding the current state of reported treatment planning by analyzing the reported incidents in our departmental ILS. Numerous other efforts demonstrate the value of ILS and reporting to improve patient safety, including the national radiation oncology incident learning system (RO‐ILS).^9^, ^21^, ^22^, ^23^, ^24^, ^25^ The reported errors were categorized, and their occurrence was continuously monitored throughout this QI effort.

#### 2.1.3 Analyze stage

In this stage, check items eligible for automation were identified and prioritized using FMEA. An itemized list of the individual physics plan check steps was compiled using: the AAPM TG‐275 draft checklist,^26^ departmental procedures, and items directly inspired by errors/near misses reported to ILS. A total of 101 physics plan check items were identified. A multidisciplinary QI team composed of radiation oncologists, physicists, dosimetrists, and therapists ranked severity and detectability of failure modes associated with these 101 plan checking steps using the TG‐100 ranking scale.^27^ The team ranked the following: (S) *Severity* of impact on patient’s radiation therapy if the error is not caught; (D) *Detectability Dormancy* as the probability of the error going undetected. *Occurrence* (O) was determined based on the records from the departmental ILS from October 2015 to October 2017. Risk priority number (RPN) was calculated for each physics plan check item using FMEA formalism:

$$RPN=Severity\left(S\right)\cdot Detectability\;Dormancy\left(D\right)\cdot Occurrence\left(O\right).$$

The plan‐checking steps were sorted in order of decreasing RPN score to determine the highest‐priority items to be addressed with the proposed script. These Pareto‐sorted check items were then evaluated for eligibility for either full or partial automation. The highest RPN‐ranked checklist items and items with severity > 7 were prioritized to be addressed by the APC tool.

#### 2.1.4 Improve stage

For the *Improve* stage an APC tool was developed as a plug‐in extension in Eclipse using an in‐house built C#‐based software within the Eclipse API. It queries the treatment plan parameters in Eclipse, executes predefined logics and rules for each check item, and outputs results and plan documentation for review. In addition to the provided Microsoft .NET class library, supplementary extensions to aid the query and verifications were added to access the data unavailable within the Eclipse API, such as the Varian ENM database (Varian Medical Systems). This allowed relational querying and reporting of ARIA R&V database information necessary to automate certain checks. Furthermore, to avoid code repetition resulting in unacceptable running time, parallel thread programming was employed together with consolidated class definitions and restricted inheritances.

**Figure 1:**
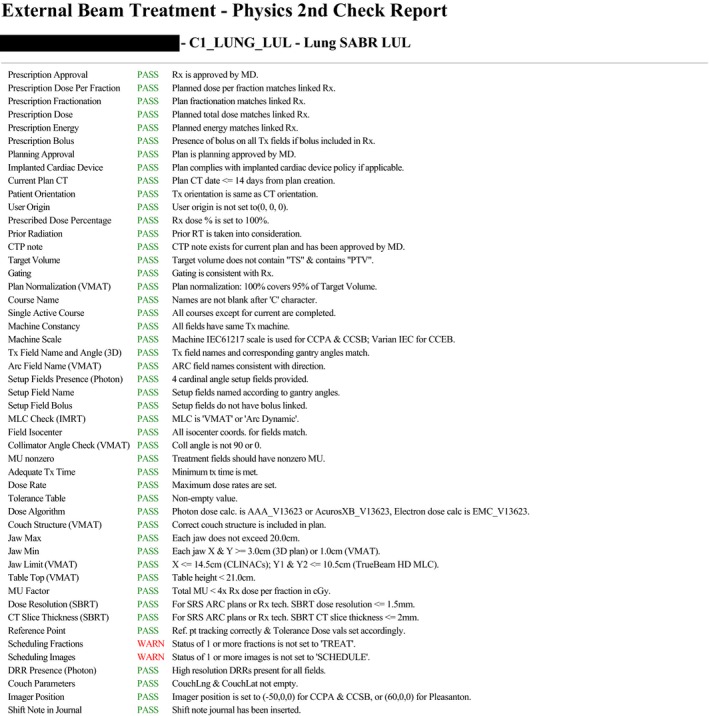
Automated plan check (APC) report interface.

The APC software was extensively tested on anonymized data sets with introduced known errors for each test unit prior to clinical release to limit false negatives (FN) and false positives (FP). The graphical user interface of the APC report is shown in Fig. 1 with each check item containing color‐coded PASS/WARN status and customized description to indicate the reason for failing a particular test. To address the highest‐ranking RPN failure mode — incorrect shift instructions for the therapists — an additional script was developed to automatically generate the shift instructions that can be easily transferred to the R&V system.

The APC script was run by dosimetrists before presenting the plan for physician’s review. If errors were caught by the APC, they were addressed, and APC was rerun until no further defects were reported. To assess the APC effectiveness in decreasing errors propagating to physics plan check, results of each APC run were saved to a database. After plan approval by physician, the physicist ran the same APC tool to verify that each physics plan check test passed before treatment approval.

#### 2.1.5 Control stage

The *Control* stage aimed to provide a sustained optimization to the APC tool by creating a feedback loop to monitor and improve the robustness of the software. An internal online feedback system based on voluntary reporting was generated and distributed to dosimetrists and physicists. Team members were encouraged to report and provide feedback as well as potential check items for automations.

The QI team conducted reviews of reported errors on a bi‐monthly basis, and actions were taken to address imminent issues and update/expand the functionality of APC.

### 2.2 Comparison of pre‐APC and post‐APC phase

Nine months post‐introduction of the APC tool, all 101 physics plan check items were re‐evaluated to update the FMEA *Occurrence* and *Detectability Dormancy* values. Pre‐ and post‐APC phases were defined as 9 months prior‐ and post‐APC implementation. A paired *t*‐test was used to determine statistical significance. Reported treatment planning errors at the time of physics plan check and therapy plan check were normalized to the total number of plans completed in the time frame and compared between the two phases. The *Six Sigma* defect‐rate‐per‐opportunity (DPO) was calculated for high severity errors occurring frequently in the pre‐APC phase, incorrect isocenter‐shift instructions and prescription/plan discrepancy, using a *Six Sigma* formalism:

$$DPO=\frac{\mathrm{Errors}}{Opportunities\;for\;Errors\;in\;a\;Plan\cdot Number\;of\;Plans}$$

This value was compared to the *Six Sigma* goal of 3.4 × 10^−06^, which was determined to be both acceptable and achievable by the QI committee. In addition, to confirm that the decrease in error frequency was attributable to the APC tool, we analyzed the database of APC output during the first and final run for each plan prior to its approval for 4 months post‐APC implementation.

### 2.3 Efficiency evaluation

To test the gain in efficiency, 9 months post‐APC introduction 20 treatment plans of three types (six VMAT, eight SBRT, and six 3D‐CRT) were prospectively stratified into two categories: APC‐assisted (three VMAT, four SBRT, and three 3D‐CRT) and manually checked (three VMAT, four SBRT, and three 3D‐CRT). For non‐APC assisted plans, dosimetrists were requested to generate the isocenter‐shift instructions manually and perform their plan preparation and plan review without initiating the APC tool. Two physicists were asked to perform the physics plan check for equal number of plan types in each category (with and without APC) and manually record the time for each check and errors detected. Two‐sample *t*‐test assuming unequal variance was used to determine statistical significance.

## 3 RESULTS

### 3.1 Failure mode and effects analysis

**Figure 2:**
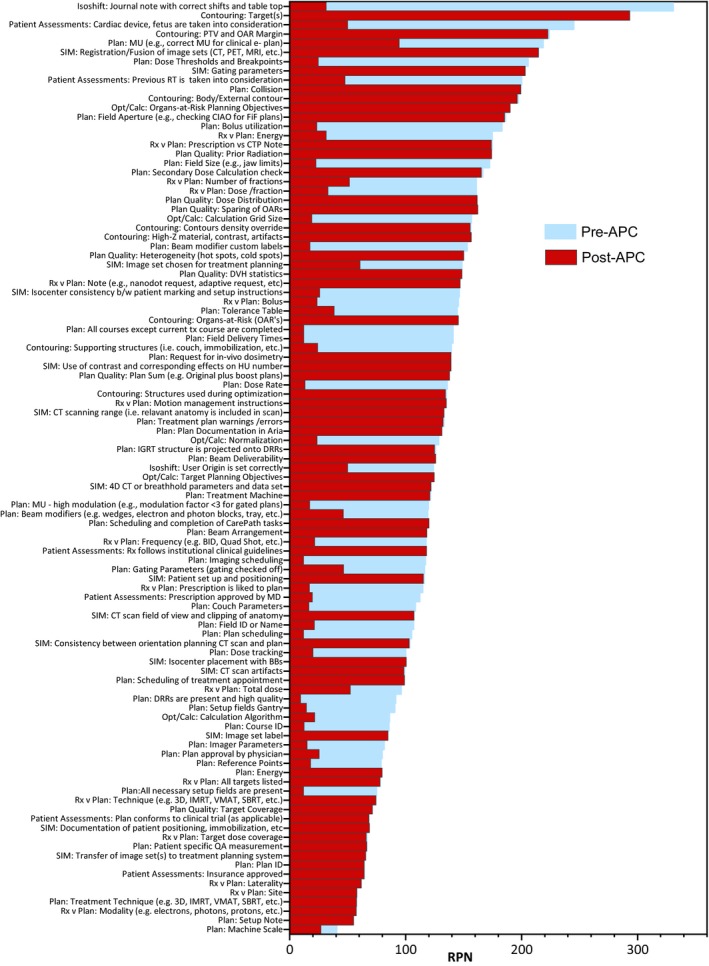
Pareto‐sorted list of failure modes of all plan check elements ranked by risk priority number value.

Overall, 101 physics plan check elements were identified. The list of plan check elements sorted by RPN is shown on Fig. 2. RPN values ranged from 40.5 to 330.8. Forty‐two elements out of 101 (41.6%) were assigned as potentially suitable for either full or partial automation within the Eclipse API environment. Among the highest‐risk items suitable for automation were: isocenter‐shift instructions provided to therapists (RPN = 330.8), cumulative items checking prescription/plan match (dose per fraction, number of fractions, energy, bolus; RPN range = 145.4–174.6), accounting for cardiac device (RPN = 245.0), dose thresholds and breakpoints (RPN = 205.7), accounting for previous radiotherapy (RPN = 200.2). Table 1 lists the checklist items with high‐severity scores (>7).

**Table 1:** Physics plan check elements with the highest severity (>7).

Physics plan check items	Severity
Rx v plan: site	9.3
Rx v plan: laterality	9.1
Contouring: target(s)	8.4
SIM: consistency between orientation of image on the CT scan and treatment plan	8.4
Rx v plan: total dose	8.4
Rx v plan: dose /fraction	8.3
Rx v plan: number of fractions	8.3
Patient assessments: cardiac device, fetus, etc. are taken into consideration for RT	8.0
Isocenter shift: user origin is set correctly	8.0
Isocenter shift: journal note with correct shifts and table top	7.9
Plan: MU (e.g., correct MU for 2D plan)	7.9
Patient assessments: previous RT taken into consideration	7.6
Plan: collision	7.3
Contouring: organs‐at‐risk (OAR's)	7.1

Abbreviations: Rx – prescription; SIM – simulation; RT – radiotherapy; MU – monitor unit.

At the time of the initial clinical release of the APC script on January 1, 2018, it contained 24 checks designed for photon 3D CRT/VMAT plans for the main cancer center. At 9 months post‐implementation and after multiple iterations of DMAIC loop, the APC script contained 89 checks to verify photon, electron, and total skin electron irradiation (TSEI) plans for the main cancer center; furthermore, it has been adapted for use at two satellite sites. Full physics plan check automation was achieved for TSEI templated treatment.

At 9 months post‐APC implementation, *Occurrence* and *Detection Dormancy* scores were re‐evaluated. The average difference between pre‐APC and post‐APC RPN values was −045.5 (range, −299.3 − 1.5). The average RPN pre‐APC was 129.2 compared to average post‐APC RPN of 83.7 (*P* < .05). Among the tests with the biggest decrease in RPN were isocenter‐shift instructions (ΔRPN = –299.3), special consideration for RT, for example, cardiac device (ΔRPN = −195.0), dose thresholds and breakpoints (ΔRPN = −181.0), and bolus utilization in the plan (ΔRPN = −159.8).

### 3.2 Frequency of reported treatment‐planning errors detected after plan approval

**Figure 3:**
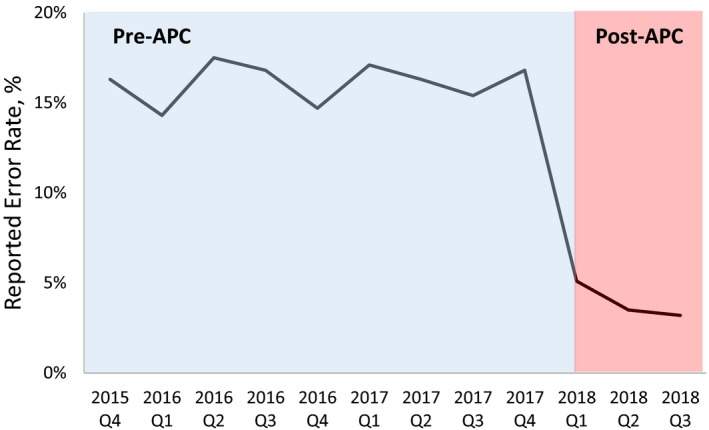
Reported treatment‐planning errors normalized to number of plans per quarter.

Figure 3 shows the frequency of reported treatment‐planning errors normalized to the total number of EBRT plans quarterly from the 4th quarter of 2015 when the ILS was introduced to the 3rd quarter of 2018. These errors were detected after the physician’s plan approval at the physics plan check, therapy plan check, and treatment. After the introduction of APC on January 1, 2018, the average frequency of reported treatment‐planning errors for the three quarters was reduced from 16.1% to 4.1%.

**Figure 4:**
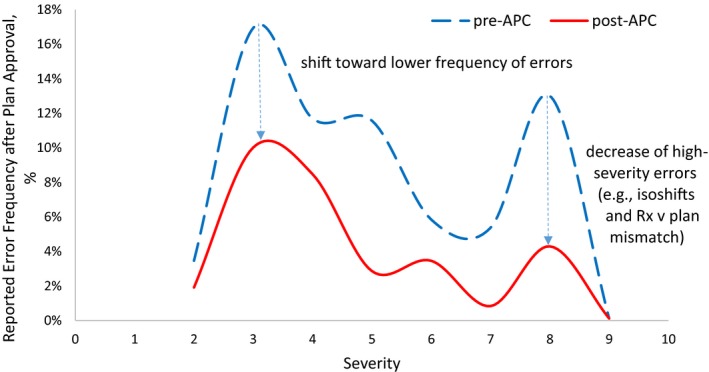
Histogram of reported errors stratified by severity and normalized by total number of errors 9 months pre‐automated plan check (APC) and 9 months post–APC.

Figure 4 illustrates the histogram of reported error frequencies normalized to the total number of errors reported and stratified by assigned severity scores (S). Evident from the histograms, the overall frequency and, particularly, the frequency of high‐severity errors, decreased in the post‐APC phase (Δ = −67% for S = 8).

**Figure 5:**
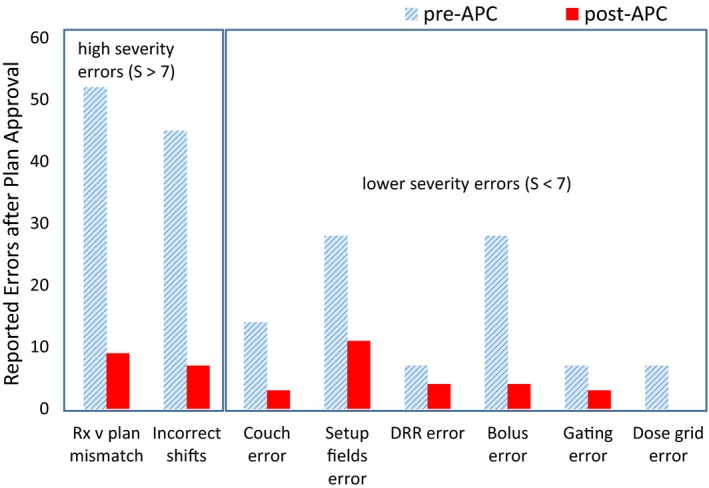
Comparison of reported treatment‐planning errors 9 months pre‐ and post‐automated plan check implementation.

The effectiveness of the APC tool is most evident in decreasing the high‐severity errors of prescription/plan mismatch and incorrect isocenter‐shift instructions (Fig. 5), which was a stated goal of the project. The total number of errors reported in the pre‐APC phase was reduced by 82.7% for prescription/plan mismatches and 84.4% for incorrect isocenter‐shift instructions. On average, all reported treatment‐planning errors decreased on by 52.9%.

The *Six Sigma* DPO was calculated for incorrect isocenter‐shift instructions and prescription/plan discrepancy for the pre‐ and post‐APC phases. For shift instruction errors, DPO was 2.60 × 10^−03^ and 2.26 × 10^−04^ for pre‐ and post‐APC phases, respectively. This indicates the shift from 4σ to 5σ process with 99.977% yield. For prescription/plan mismatch errors, the process stayed within 4σ error rate with 3.27 × 10^−03^ and 8.41 × 10^−04^ DPO values for pre‐ and post‐APC phases, respectively.

### 3.3 Frequency of detected treatment‐planning errors prior to plan approval

**Figure 6:**
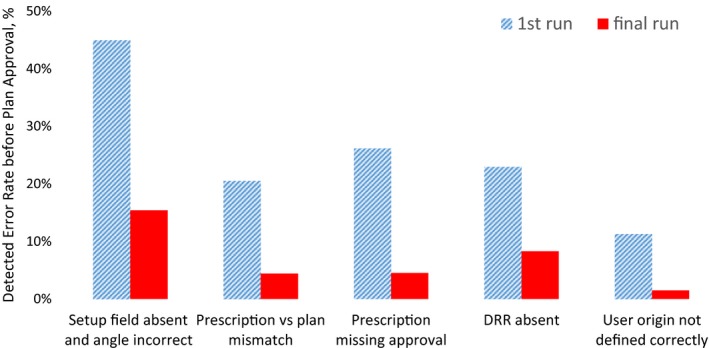
Comparison of the most frequent errors between the first and final automated Plan Check (APC) run for all plans for 4 months post‐APC implementation.

To verify if the decrease in error frequency is attributed directly to the APC tool, the database of APC output was analyzed for the first and final run for each plan prior to its approval. Figure 6 illustrates the comparison between the outcome from the first and final APC run for the top 6 high‐occurrence errors, collected within 4 months post‐APC introduction. The error rates, that is, number of failed checks over number of total checked items, dropped from 13.3% to 4.5% between the first and final APC executions.

### 3.4 Efficiency improvements

Twenty clinical treatment plans (eight SBRT, six VMAT, and six 3D‐CRT) were prospectively assigned to either a manual physics plan check or the APC‐assisted plan check. Five errors were found in 10 manually checked plans, including high‐severity errors of prescription/plan discrepancy and missing shift note. In contrast, only one error propagated to physics plan check out of the 10 APC‐checked plans demonstrating an 80% error decrease. On average, it took 21.7 ± 5.9 min to perform plan check manually vs 11.1 ± 8.6 min to perform physics plan check with APC assistance. The average physics plan check time decrease with APC assistance was 10.1 ± 7.3 min. (*P* = .005) per plan.

## 4 DISCUSSION

From this analysis, it is evident that despite the absence of medical events, the EBRT process was susceptible to high‐severity errors. These high‐risk items were prioritized for automation: for example, the separate shift note script was created to generate the note automatically to be copied/pasted into the Journal Note in Aria. Not all the items were eligible for automation, for example, verification of “contouring targets” item with severity of 8.4 was best suited for physician‐peer review either during preplanning contour review or pretreatment Chart Rounds review.

In our study, analysis of the rate of reported treatment‐planning errors after plan approval comparing 9 months pre‐ and post‐APC implementation demonstrated a decrease from 16.1% to 4.1% with the introduction of the APC tool. Holdsworth et al. reported the overall decrease in total plan revisions from 18% to 11.2% after introduction of their in‐house automatic plan checking software.^10^ Covington et al. reported 60% reduction in the number of patient delays in the 6 months after their in‐house plan check tool implementation.^14^

We also observed substantial improvements in reduction of high‐severity errors. An FMEA RPN ranking re‐evaluation 9 months post‐APC demonstrated a statistically significant average decrease in RPN values from 129.2 to 83.7 (*P* < .05), suggesting a safer external beam treatment‐planning/delivery practice. A histogram of reported errors stratified by severity demonstrated the shift toward decreased frequency of errors, most importantly for higher‐severity errors. For shift instruction errors, the shift from 4σ to 5σ process with 99.977% yield was observed in the post‐APC phase. For prescription/plan mismatch errors, the process stayed within 4σ error rate. The calculation of the overall DPO was not attempted as opportunities for error are highly plan specific depending on type of plan, beam number, number of OARs, targets, etc.

To eliminate the inherent uncertainty stemming from stochastic nature of incidence reporting and to separate the influence of other quality improvement processes (staff training, new policy/workflow enforcement) on the decrease in reported error after introduction of the APC tool, we analyzed the database of the APC output during the first and final run for each plan prior to its approval during 4 months after introduction of the APC tool. The error rates, that is, number of failed checks over number of total checked items, dropped from 13.3% to 4.5% since the first run. This decrease in detected errors indicates that planners were alerted to review/revise the errors before plan finalization, which effectively prevented the error propagation downstream. However, this effectiveness estimation is dependent on the behavior of the planner and his/her reliance on the APC to catch the errors rather than manually preparing the plan for approval and running the APC once before the physician’s review. The presence of errors after the final APC run signify either existence of FP, the planners not re‐running the APC after rectifying the errors or planner not addressing the errors. The latter was prevalent in the early post‐APC phase when dosimetrists were adjusting to reliance on the APC tool to detect the defects. The feedback loop was instrumental in getting dosimetrists’ support in this project: they were actively participating in reporting false positives, suggesting new tests and reviewing the incident reports on bi‐monthly basis.

Our efficiency study carried out by prospectively stratifying 20 patient plans into two categories: manual and APC‐assisted plan preparation/check, showed a statistically significant decrease in physics plan check time (~10 min) and decrease in errors propagating to physics plan check (80%). Other institutions reported an average of 2–5 min time saving associated with the use of the automated plan checking tools.^10^, ^14^ The decrease in errors propagating to physics plan check due to the APC may be related to the observed gain in efficiency since physicists did not have to spend time correcting the errors. The physics plan check time decrease provided by automation will allow the physicist to spend more time in evaluating plan's overall quality.

Our analysis is not without known limitations. Incidence reporting cannot be assumed to be consistent throughout the time period or complete. The environment is certainly not controlled as policies and procedures get introduced. We would like to note though that addition of four new dosimetrists (40%) to the team during the post‐APC phase still resulted in decrease in errors compared to pre‐APC phase. In addition to the above uncertainties, the FMEA is a semiquantitative analysis and is highly dependent on the users’ assessment of the risk factors and their impact in the clinic.

A logical next step in improvement is converting APC checks into forcing the user to correct the errors, not merely detecting them. In addition, apart from rule‐based automated checking approaches, knowledge‐based automated QA/QC methods have recently shown great potential in decision‐making in radiotherapy.^28^, ^29^, ^30^ They can be applied to detect outliers, raise warnings on suboptimal plans, ensure optimal dose prescription and treatment plan quality, and to predict treatment outcomes.^31^, ^32^, ^33^ Incorporating knowledge‐based methods, in combination with current rule‐based APC software, will be explored in the future work.

## 5 CONCLUSIONS

In this work, a *Six Sigma* DMAIC‐driven QI project conducted in our radiation oncology department was described and demonstrated to be effective in decreasing errors stemming from treatment planning and improving the efficiency of the physics plan check process. This work shows that rule‐based automation can have a significant impact on the efficiency and quality of radiation oncology treatments. We hope these results encourage other radiation oncology departments to consider incorporating *Six Sigma* methodology to create and implement a custom‐made treatment plan checking software in their clinical practice. We will be glad to share our experience with creating and implementing the APC tool in the clinic.

## CONFLICT OF INTEREST

No conflict of interest for all authors except Lei Xing, PhD: Research grant from Varian Medical Systems, Palo Alto, CA

## AUTHORS RESPONSIBLE FOR STATISTICAL ANALYSES

Shi Liu and Nataliya Kovalchuk
